# Functional Trait Divergence in Feeding Ecology Between Two Closely Related *Upeneus* Species in the Beibu Gulf: An Ecological Morphology Analysis

**DOI:** 10.1002/ece3.72331

**Published:** 2025-10-15

**Authors:** Jiawei Fu, Xiaodong Yang, Konglan Luo, Zhisen Luo, Jingxi Wang, Bin Kang, Xiongbo He, Yunrong Yan

**Affiliations:** ^1^ College of Fisheries Guangdong Ocean University Zhanjiang China; ^2^ College of Fisheries China Ocean University Qingdao China; ^3^ Guangdong Provincial Engineering and Technology Research Center of Far Sea Fisheries Management and Fishing of South China Sea Guangdong Ocean University Zhanjiang China

**Keywords:** ecomorphology, feeding ecology, functional traits, mechanisms of coexistence, niche differentiation

## Abstract

Closely related species coexisting in the same habitat often undergo niche differentiation or morphological adaptations to reduce interspecific competition and secure sufficient resources, particularly when their ecological niches overlap. Such differentiation may manifest as niche expansion or enhanced morphological adaptations to improve competitive advantages in resource utilization. This study focused on *Upeneus sulphureus* and *Upeneus tragula* in the Beibu Gulf, using functional trait analysis, stable isotope techniques, and morphological comparisons to explore their functional niche differentiation and resource utilization strategies. Functional richness analysis showed that *U. tragula* had significantly higher functional richness (22.33%, *n* = 38) than *U. sulphureus* (11.64%, *n* = 67), with low functional niche overlap (27.91%), indicating significant divergence in resource utilization. Trophic niche analysis further revealed that *U. tragula* had significantly higher trophic diversity (CD) and core niche breadth (SEAc) than *U. sulphureus* , indicating broader resource utilization capabilities and adaptability. The results revealed significant differences in functional traits between *U. sulphureus* and *U. tragula* : *U. sulphureus* has a robust body, longer pectoral fins and snout, and a larger oral gape, exhibiting lower δ^13^C (−18.38‰ ± 0.35‰) and δ^15^N (13.31‰ ± 0.47‰) values (*p* < 0.05), primarily feeding on cephalopods. In contrast, *U. tragula* has a streamlined body, larger eye diameter, and longer barbels, displaying higher δ^13^C (−15.23‰ ± 0.96‰) and δ^15^N (15.36‰ ± 0.38‰) values (*p* < 0.01), primarily preying on small benthic invertebrates. These morphological differences are closely related to the feeding strategies and habitat preferences of the two goatfish species, reflecting their ecological adaptive divergence. This study reveals that *U. sulphureus* and *U. tragula* achieve coexistence through functional trait and trophic niche differentiation, providing important insights into species adaptive evolution and resource allocation mechanisms in ecosystems.

## Graphical abstract

This research not only provides new insights into the niche differentiation and adaptive evolution of closely related species but also offers significant evidence for understanding resource allocation mechanisms within ecosystems.

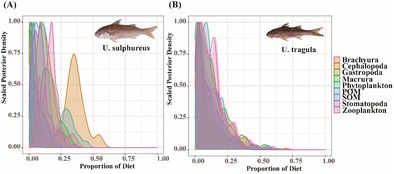

## 1 Introduction

Functional traits are central to the study of ecomorphology, encompassing both morphometric data (Collar and Wainwright 2009; Keppeler et al. 2020; Langerhans et al. 2013; Webb 1994) and categorical variables related to foraging behavior, feeding strategies, trophic levels, body size, locomotion, migratory capacity, life habits, activity ranges, and habitat distribution (Farré et al. 2016; Halpern and Floeter 2008; Somerfield et al. 2008). Ecomorphology, through the analysis of these functional traits, reveals the close relationship between species' morphology and their ecological functions, providing critical insights into how species adapt to their environments and utilize resources (Aneesh Kumar et al. 2017). A significant advantage of the ecomorphological approach based on functional traits lies in its ability to achieve broader generalizations across systems, as functional traits can transcend different ecosystems, even when these systems do not share the same species (Mindel et al. 2016). This method serves as a powerful tool for investigating niche differentiation, resource utilization strategies, and interspecific interactions within ecosystems. A study by Aneesh Kumar et al. (2017) on seven deep‐sea fish species in the southeastern Arabian Sea demonstrated that functional divergence minimizes niche overlap, facilitating coexistence under resource constraints.

Although ecomorphological methods hold significant value, they are not without limitations. For instance, species with similar morphological traits may achieve coexistence through temporal partitioning (Colmenero et al. 2010; Gutman and Dayan 2005) or by utilizing different ecological niches at various life stages (Cucherousset et al. 2011; Skulason and Smith 1995; Zhao et al. 2014). Additionally, species may capture the same type of prey using alternative functional traits (Wainwright et al. 2005) or employ different hunting strategies, where trade‐offs between these strategies allow for competitive coexistence even under shared resource conditions. These complexities highlight that while ecomorphology provides an important framework for studying species adaptation and coexistence mechanisms, a comprehensive understanding of species coexistence and resource utilization requires the integration of other ecological factors for a more holistic analysis.

Research on fish coexistence and competition primarily focuses on interspecific relationships (Pratchett 2005), while studies on closely related species remain limited (Pol et al. 2023). Closely related species exhibit high overlap in resource use due to their similar morphology and ecological habits (Liu et al. 2015). According to the limiting similarity theory (Macarthur and Levins 1967), species with excessive similarity competitively exclude one another, explaining why even minor functional differences exist between coexisting species. These differences reflect species‐specific adaptations to resources, habitats, or environmental conditions. Niche differentiation—through spatial, temporal, or resource‐based partitioning—enables the formation of distinct ecological niches. This differentiation is considered a key mechanism for species coexistence (Amarasekare 2003; Kylafis and Loreau 2011; Schoener 1974). Functional morphology provides an effective framework for analyzing and comparing phenotypic traits related to feeding and locomotion (Bertossa 2011). Ecological studies based on species' functional traits—those that influence organismal function and, by extension, ecosystem function—are regarded as a powerful approach to deciphering the critical functional roles of species within ecosystems (B. H. Walker 1992, 1995).

Research on the functional traits of fish feeding has been gradually increasing. Gosline (1984) conducted a systematic study on the body structure and functions of Mullidae, with a particular focus on their morphological characteristics, especially the detailed description of barbel structures. This study revealed the critical role of barbels in food acquisition, intraspecific communication, and environmental adaptation. McCormick (1993) further investigated the developmental changes in barbel structure during the settlement phase of *U. tragula* , elucidating the importance of barbel structure for ecological adaptability and its dynamic changes during ontogeny. Uiblein et al. (1998) employed morphometric methods to quantitatively analyze interspecific and intraspecific morphological variations within the genus *Upeneus*, uncovering the functional relationships between morphological differences and resource utilization. Additionally, Mittelheiser et al. (2022) combined morphological and stable isotope analyses to explore the ecomorphological partitioning of six goatfish species in Madagascar's coral reefs, identifying head traits as key features of feeding functionality in Mullidae. Despite these studies providing important insights into the ecological significance of fish feeding functional traits, there remains a lack of in‐depth research on the differences between closely related species. The present study aims to address three fundamental research questions: (1) What are the significant divergences in feeding functional traits between the two *Upeneus* species? (2) How do these phenotypic differences reflect their ecological adaptations and resource utilization strategies? (3) Can analysis of these feeding‐related functional trait variations elucidate the evolutionary implications of niche differentiation and coexistence mechanisms between these congeneric species? Therefore, a deeper exploration of the differences in feeding functional traits among closely related species and their ecological implications is of great significance for understanding species coexistence mechanisms and ecological adaptations.

*U. sulphureus* and *U. tragula* both belong to the Mullidae family and the genus *Upeneus* (Uiblein and Heemstra 2010). They are important fish species with relatively stable populations in the Beibu Gulf, occupying key ecological niches (Liang‐liang et al. 2023). Due to their high morphological similarity and overlapping distribution ranges (Mohamed and Resen 2010; Uiblein and Gouws 2014; Uiblein and Heemstra 2011), there is potential for interspecific competition between them. This study focuses on these two coexisting *Upeneus* species in the Beibu Gulf, employing functional trait analysis and carbon‐nitrogen stable isotope techniques to systematically evaluate the separation and overlap of their functional and trophic niches. The aim is to reveal the differentiation in functional traits and feeding strategies between the two species. The research emphasizes the differences in resource utilization, habitat selection, and survival strategies between *U. sulphureus* and *U. tragula* , thereby elucidating the mechanisms and patterns of their niche differentiation. By analyzing their feeding functional traits and their relationship with stable isotopes, this study not only contributes to understanding the coexistence mechanisms of closely related species in similar habitats but also provides a scientific basis for predicting how fish functional morphology responds to feeding differences.

## 2 Materials and Methods

### 2.1 Sample Collection and Processing

In 2022, this study conducted fixed‐point bottom trawl surveys in the Beibu Gulf, collecting various samples including seabed sediment organic matter (SOM), suspended particulate organic matter (POM), phytoplankton, zooplankton, aquatic invertebrates, and fish. To further enhance the data, supplementary sampling was conducted in the spring of 2024 at fishing ports around the Beibu Gulf (Beihai Qiaogang Fishing Port in Guangxi and Baimajing Central Fishing Port in Hainan). A total of 105 specimens were collected, comprising: *U. sulphureus* (*n* = 67) with total lengths ranging 138–181 mm (160.36 ± 9.71 mm), *U. tragula* (*n* = 38) exhibiting total lengths of 121–161 mm (141.95 ± 8.79 mm). All samples were measured for biological parameters on‐site according to the Specifications for Oceanographic Survey, including body length, body weight, gonad weight, and gutted weight, followed by rapid freezing at low temperatures and transportation to the laboratory.

In the laboratory, all specimens underwent systematic taxonomic identification and comprehensive biological measurements. Species identification was conducted following the standardized classification system established by the Food and Agriculture Organization of the United Nations (Fischer and Hureau 1985). Given that animal tissues (e.g., muscle) can reflect long‐term dietary information (McIntyre and Flecker 2006; Qu et al. 2019), we collected tissue samples from different groups: white dorsal muscle from fish, abdominal muscle from shrimp, muscle from the first cheliped of crabs, arm muscle from cephalopods, shelled muscle from snails, and adductor muscle from bivalves. For other smaller invertebrates lacking sufficient white muscle tissue, the entire individual was used for stable isotope analysis. Prior to δ^13^C analysis of SOM samples and whole small crustaceans, the samples were treated to remove the influence of inorganic carbon (Post 2002). Specifically, the isotope samples were divided into two portions: one treated with 1 mol/L HCl to dissolve CaCO_3_ for δ^13^C analysis, and the other used directly for δ^15^N analysis (Kanaya et al. 2007). Given that single‐celled phytoplankton absorb nutrients through non‐selective phagocytosis, this study conducted a comprehensive analysis of mixed phytoplankton samples composed of multiple species.

### 2.2 Data Acquisition

#### 2.2.1 Morphological Data

Food acquisition and locomotion, as critical survival functions, typically require the coordinated involvement of multiple organs (Arreola and Westneat 1996; Mouillot et al. 2013). To describe the morphological characteristics of fish and reflect their functional adaptations (Albouy et al. 2011; Villéger et al. 2008, 2010; Zhao et al. 2014), this study measured 12 morphometric traits: standard length (Sl), head depth along the vertical axis of the eye (Hd), body depth (Bd), caudal peduncle minimal depth (CPD), maximal caudal fin depth (CFD), pectoral fin length (PFl), eye diameter (Ed), distance from the top of the mouth to the bottom of the head along the head depth axis (Mo), mouth depth (Md), mouth width (Mw), barbel length (BaL), and snout length (SnL); all morphometric measurements were collected using digital vernier calipers (precision: 0.01 mm) to ensure data accuracy and reproducibility. These measurements were integrated into nine functional indices (Table 1), including traits closely related to food acquisition: oral gape shape (MS = Md/Mw), oral gape position (MP = Mo/Hd), barbel length (BAL), snout length (SNL), and relative eye diameter (RED = Ed/Hd); and traits related to locomotion: relative head depth (RHD = Hd/Bd), relative body width (RBW = Bd/Sl), relative pectoral fin length (RPFL = PFl/Sl), and caudal peduncle throttling (CPT = CFD/CPD) (Albouy et al. 2011; Villéger et al. 2010). These indices provide a comprehensive framework for understanding the functional morphology and ecological adaptations of the studied fish species.

**TABLE 1:** List of the 9 functional traits associated with food acquisition and locomotion (adapted from Villéger et al. 2010). The letter in brackets indicates the function associated with each trait (F, food acquisition and L, locomotion). Coefficients of variation (CV) measured in the population.

Functional traits	Measure	Ecological meaning	CV%
Oral gape shape (F)	Md/Mw	Prey shape and food acquisition	13.07
Oral gape position (F)	Mo/Hd	Position of prey in the water	19.72
Eye diameter (F)	Ed/Hd	Prey detection	10.93
Relative head depth (L)	Hd/Bd	Maneuverability and position in the water column	8.32
Relative body width (L)	Bd/Sl	Vertical turning ability	11.39
Relative pectoral fin length (L)	PFl/Sl	Maneuverability at low speeds	14.17
Caudal peduncle throttling (L)	CFd/CPd	Swimming endurance	24.81
Barbel length (F)	BaL	Prey detection	15.60
Snout length (F)	SnL	Digging depth	13.59

Abbreviations: BaL, barbel length; Bd, body depth; CFd, maximal caudal fin depth; CPd, caudal peduncle minimal depth; Ed, eye diameter; Hd, head depth along the vertical axis of the eye; Md, mouth depth; Mo, distance from the top of the mouth to the bottom of the head along the head depth axis; Mw, mouth width; PFl, pectoral fin length; Sl, standard body length; SnL, snout length.

#### 2.2.2 Stable Isotope Data

The muscle samples were placed in the sample tray of a freeze dryer (Christ, Alpha1‐4/2‐4LD Plus, Germany) and freeze‐dried at −48°C for 48 h until the muscle reached a constant weight. The samples were then removed, and two small steel beads were added to each centrifuge tube containing the sample. The tubes were placed in a bead homogenizer (BIOSPEC MiniBeadbeater‐16, USA) and ground for 1 min. The ground muscle powder was encapsulated and sent to the Isotope Laboratory of the College of Fisheries at Guangdong Ocean University for analysis. The samples were measured using an EA Isolink CN/HO elemental analyzer and a Delta V Advantage stable isotope ratio mass spectrometer.

The stable isotope ratios were expressed using the internationally recognized δ notation, calculated as follows:

$$\mathrm{\delta X}=\left(\frac{R\text{sample}}{R\text{standard}}-1\right)\times 1000$$

where *δX* represents the stable isotope ratio of carbon (δ^13^C) or nitrogen (δ^15^N), *R*sample is the ratio of carbon or nitrogen stable isotopes in the sample, and *R*standard is the ratio of the standard reference material. For carbon isotopes, the standard is Pee Dee Belemnite (PDB), and for nitrogen isotopes, the standard is atmospheric nitrogen (AIR). The carbon isotope ratio is expressed as ^13^C/^12^C, and the nitrogen isotope ratio is expressed as ^15^N/^14^N.

#### 2.2.3 Data on Potential Food Sources

Based on the research conducted by our team on the stomach contents of *U. sulphureus* and *U. tragula* , as well as referencing literature on the dietary habits of these two species in the same sea area, the potential food sources were identified (Golani and Galil 1991; Kolasinski et al. 2009; Ramteke et al. 2015). The potential prey organisms for these species may include Brachyura, Cephalopoda, Gastropoda, Macrura, Phytoplankton, Zooplankton, Stomatopoda, Particulate Organic Matter (POM), and Soil Organic Matter (SOM).

According to the principle that the carbon and nitrogen stable isotope compositions of predators align with those of their prey, the stable isotope ratios of *U. sulphureus* , *U. tragula* , and the various prey categories were selected. The MixSIAR model was then used to analyze the proportional contributions of each prey category to the diet of the predators. This approach helps to understand the relative importance of different prey organisms in the food sources of *U. sulphureus* and *U. tragula* .

### 2.3 Statistical Analysis of Data

To ensure that each functional trait was given equal weight, the data for the nine functional traits were standardized using deviation standardization (mean = 0, variance = 1) prior to statistical analysis. Based on the standardized functional traits of each individual, Principal Component Analysis (PCA) was employed to construct a multidimensional functional niche, and principal components with eigenvalues greater than 1 were selected as the primary axes. The functional identity (FIde), defined as the abundance‐weighted average of functional traits, was calculated for each species using the following formula:

$$FI\text{d}e=\overset{n}{\underset{i=1}{\sum }}Pi\times ti$$

where *P*_*i*_ represents the relative abundance of species *i* in the community, *n* is the number of species, and *t*_*i*_ denotes the functional trait value of species *i*. To test for significant differences in functional identity between the two species, a non‐parametric permutational multivariate analysis of variance (PERMANOVA) was conducted. This method assesses whether the observed differences in functional traits between species are statistically significant, providing insights into their ecological differentiation.

Next, the Quickhull algorithm was employed to calculate the actual functional richness (i.e., the size of the functional niche, represented by the volume of the convex hull formed by all individuals of each species in the functional niche space) for both species, as well as the actual degree of functional niche overlap between them. The functional niche overlap (FOve) was calculated based on the method proposed by Villéger et al. (2013). The percentage of shared functional richness between the two populations was determined using the following formula:

$$\text{FOve}=\text{FRic}\left(\mathrm{Sn}\cap \mathrm{Sm}\right)/\left(\text{FRic}\left(\mathrm{Sn}\right)+\text{FRic}\left(\mathrm{Sm}\right)‐\text{FRic}\left(\mathrm{Sn}\cap \mathrm{Sm}\right)\right)$$

where FRic(Sn) is the volume of the convex hull occupied by species *n*, FRic(Sm) is the volume of the convex hull occupied by species m, and FRic(Sn∩Sm) is the volume of the intersection of the convex hulls occupied by both species. A functional niche overlap value closer to 0 indicates lower functional similarity between the two species, while a value closer to 1 indicates higher functional similarity. This metric provides a quantitative measure of the degree to which the functional niches of the two species overlap, reflecting their ecological differentiation and resource utilization strategies.

The Bayesian mixing model, incorporating stable isotopes of consumers and potential food sources, was run using the “MixSIAR” package (chainLength = 1000, burn = 500, thin = 1, chains = 3, calcDIC = TRUE) in R (version 4.1.3) to estimate the contribution rates of different food sources to the consumers (Moraes and Henry‐Silva 2018). In the model, the enrichment factor for δ^13^C was set to 1.0‰ ± 0.4‰ (Zhao et al. 2022), while the enrichment factor for δ^15^N was based on the proportional trophic framework proposed by Hussey et al. (2014), which integrates experimental data and meta‐analysis. The average enrichment factor for each prey category was used, calculated as follows:

$$\Delta^{15}N=5.92-0.27P$$

where Δ^15^N is the enrichment factor, and *P* is the δ^15^N value of the consumer.

The SIBER package in R was used to plot the δ^13^C and δ^15^N niche space for the two *Upeneus* species and calculate ecological metrics including: (1) Centroid Distance (CD), representing the overall dietary displacement of populations; (2) δ^13^C Range (CR), reflecting the diversity of carbon sources; (3) δ^15^N Range (NR), indicating the breadth of trophic levels exploited; (4) Mean Nearest Neighbor Distance (MNND), quantifying intraspecific competition intensity; (5) Standard Deviation of Nearest Neighbor Distance (SDNND), characterizing dietary variation within populations; (6) Standard Ellipse Area corrected (SEAc), describing core niche width; and (7) Total Area (TA), measuring the complete isotopic niche space. Collectively, these complementary metrics provide a comprehensive understanding of resource partitioning strategies and niche differentiation patterns between the two sympatric species. Statistical analysis confirmed that δ^13^C and δ^15^N values for both species followed normal distributions (Shapiro–Wilk test, all *p* > 0.05), but exhibited heteroscedasticity (Levene's test, *p* < 0.05). Consequently, interspecific differences in isotopic ratios were assessed using nonparametric Kruskal‐Wallis tests. Pearson correlation analysis was conducted to examine the relationships between species' functional traits and δ^13^C and δ^15^N ratios. All calculations and statistical analyses were performed using R and IBM SPSS Statistics 24 software.

## 3 Results

### 3.1 Functional Niche Based on Functional Traits

The total length ranges of *U. sulphureus* (*n* = 67) and *U. tragula* (*n* = 38) were 138–181 mm (160.36 ± 9.71 mm) and 121–161 mm (141.95 ± 8.79 mm), respectively. To minimize intraspecific variation, individuals of similar sizes were selected. High interspecific variation was observed across all nine functional traits. The caudal peduncle throttling exhibited the highest coefficient of variation (CV) at 24.81%, while relative head depth showed the lowest CV at 8.32%. The CVs for other traits were as follows: oral gape shape (13.07%), oral gape position (19.72%), eye diameter (10.93%), relative body width (11.39%), relative pectoral fin length (14.17%), barbel length (15.60%), and snout length (13.59%). The average coefficient of variation for all functional traits was 14.62% ± 4.69% (Table 1).

In the PCA analysis, the first four principal components (PCs) had eigenvalues greater than 1 and collectively explained 82.1% of the total variance (PC1 = 41.3%, PC2 = 17.7%, PC3 = 15.1%, PC4 = 8.0%; Table 2). Specifically, PC1 was primarily driven by functional traits related to locomotion. As PC1 values increased, individuals exhibited stronger vertical turning ability and maneuverability (pectoral fins positioned more anteriorly relative to the body) but weaker endurance (thinner caudal peduncle). PC2 was mainly associated with functional traits related to food acquisition. Higher PC2 values corresponded to individuals with more ventrally inclined mouths, larger oral gaps, larger eyes, and eyes positioned closer to the top of the head. PC3 was primarily linked to functional traits involved in food detection. As PC3 values increased, individuals had longer barbels.

**TABLE 2:** Pearson correlation coefficients between the four principal components analysis axes and the 9 functional traits.

Functional traits	PC1 (41.3%)	PC2 (17.7%)	PC3 (15.1%)	PC4 (8.0%)
MS	**0.37**	**0.56**	**0.37**	**0.20**
MP	**0.51**	**0.42**	−0.15	**0.64**
RED	**−0.72**	**0.48**	0.17	0.07
RHD	**−0.41**	**−0.45**	**−0.61**	**0.36**
RBW	**0.91**	0.19	0.08	**−0.25**
RPFL	**0.81**	0.12	**−0.43**	−0.06
CPT	**−0.81**	0.01	**0.26**	0.08
BAL	**0.26**	**−0.52**	**0.72**	**0.25**
SNL	**0.65**	**−0.59**	0.15	0.13

*Note:* Significant *p*‐values are in bold.

Abbreviations: BAL, barbel length; CPT, caudal peduncle throttling; MP, oral gape position; MS, oral gape shape; RBW, body width; RED, relative eye diameter; RHD, relative head depth; RPFL, relative pectoral fin length; SNL, snout length.

**FIGURE 1:**
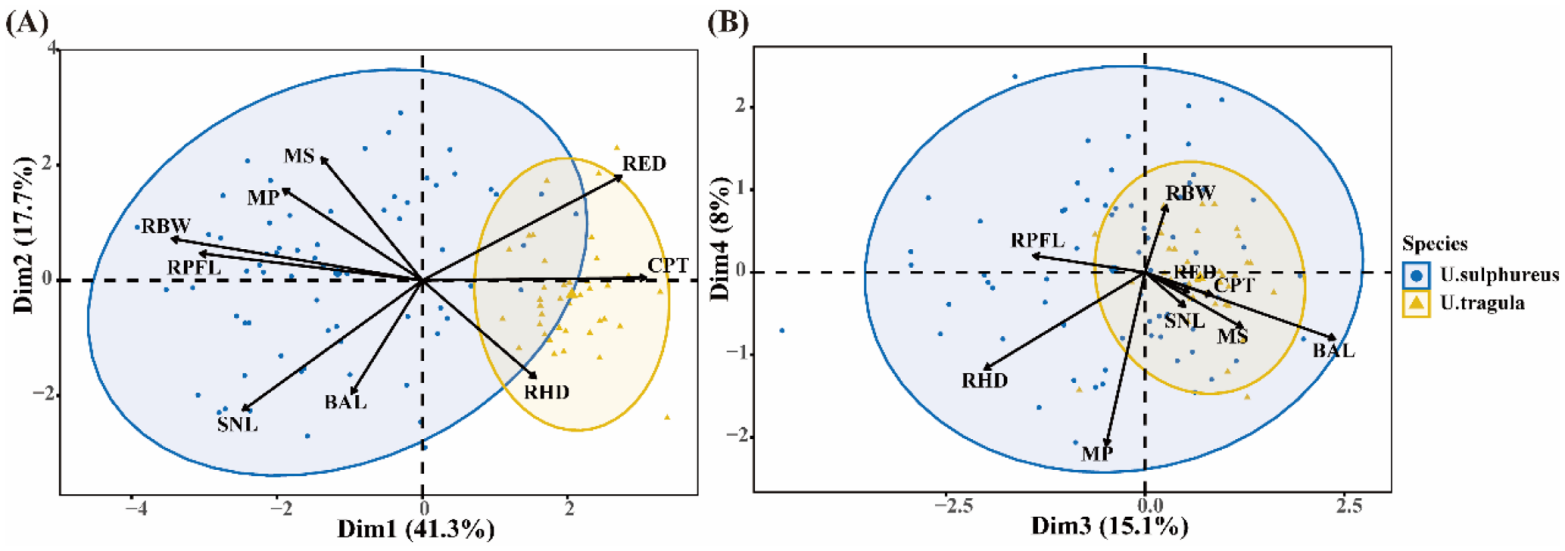
Distribution of *U. sulphureus* and *U. tragula* in functional niches. BAL, barbel length; CPT, caudal peduncle throttling; MP, oral gape position; MS, oral gape shape; RBW, body width; RED, relative eye diameter; RHD, relative head depth; RPFL, relative pectoral fin length; SNL, snout length. PCA of morphological traits with convex hulls and confidence ellipses. Blue circles: *U. sulphureus* ; yellow triangles: *U. tragula* . Arrow length indicates correlation strength.

Analysis based on functional traits revealed significant differences in functional niche characteristics between *U. sulphureus* and *U. tragula* . The measured functional richness of *U. sulphureus* was 11.64% (*n* = 67), significantly lower than that of *U. tragula* at 22.33% (*n* = 38), with a functional niche overlap of 27.91% between the two species (Figure 1). The PCA results showed distinct spatial distributions in their functional niches (Table 3), with *U. sulphureus* primarily located in the negative region of PC1, characterized by a robust body, longer pectoral fins, a longer snout, and a larger oral gape. In contrast, *U. tragula* were mainly distributed in the positive regions of PC1 and PC3, displaying a streamlined body shape, larger eye diameter, slender barbels, and a wider caudal peduncle.

**TABLE 3:** Loadings of the first four principal components for nine functional traits.

Functional traits	PC1	PC2	PC3	PC4
MS	−0.19	0.45	0.32	−0.24
MP	−0.26	0.33	−0.13	−0.75
RED	0.38	0.38	0.14	−0.09
RHD	0.21	−0.35	−0.52	−0.42
RBW	−0.47	0.15	0.07	0.29
RPFL	−0.42	0.10	−0.36	0.07
CPT	0.42	0.01	0.22	−0.10
BAL	−0.13	−0.41	0.62	−0.29
SNL	−0.34	−0.47	0.13	−0.15

### 3.2 Trophic Niche Based on Stable Isotopes

**FIGURE 2:**
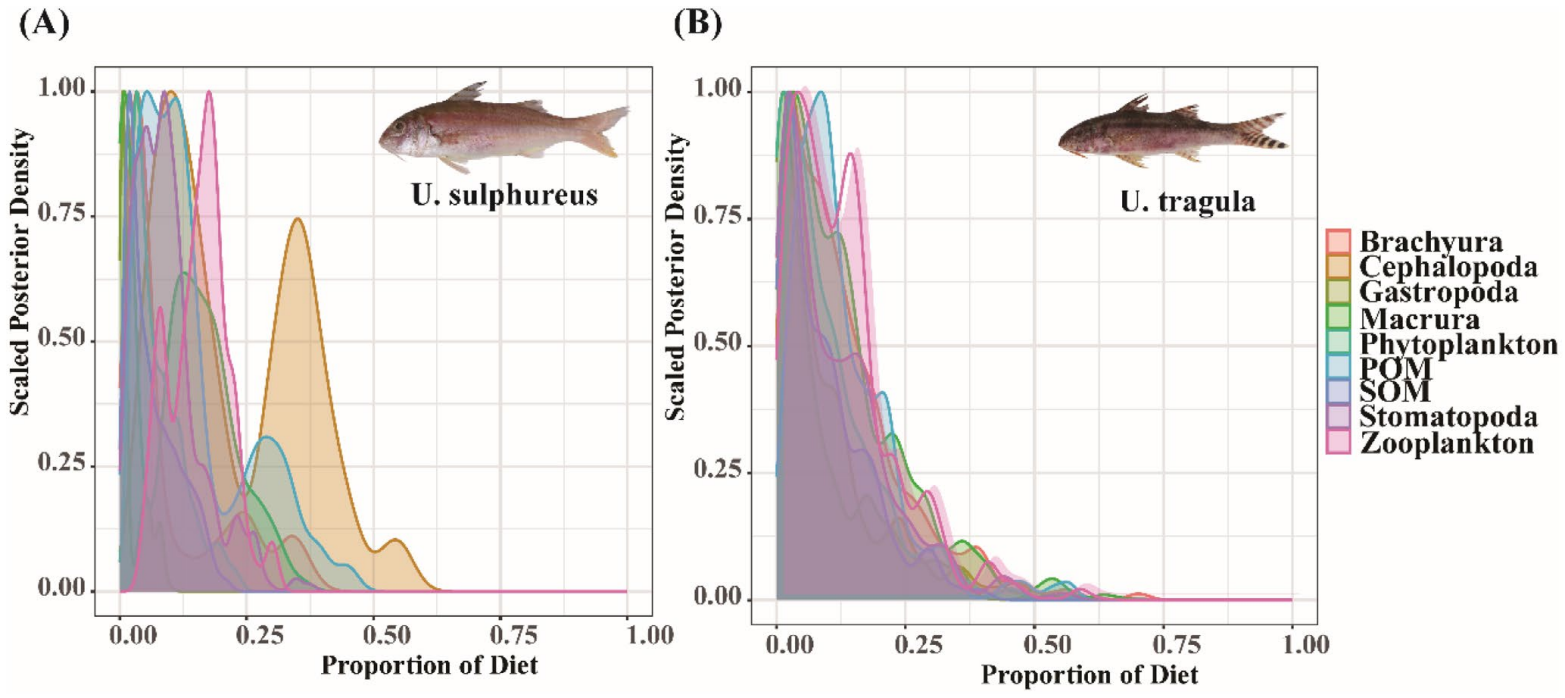
MixSIAR analysis results of dietary source contributions for two *Upeneus* species in the Beibu Gulf. (A) Probability density distribution of source contributions to *U. sulphureus* ; (B) Probability density distribution of source contributions to *U. tragula* .

The stable isotope results (Table 4) showed that the δ^13^C values of potential food sources ranged from −26.32 to −8.16, while δ^15^N values ranged from 2.55 to 16.25, indicating a wide variation. Significant differences were observed in the δ^13^C (*p* < 0.001) and δ^15^N (*p* < 0.001) values between *U. sulphureus* and *U. tragula* . Specifically, *U. tragula* exhibited higher δ^13^C and δ^15^N values compared to *U. sulphureus* . Since δ^15^N values are enriched differently across trophic levels, these results suggest that the two species have distinct food sources. Based on the MixSIAR model analysis (Figure 2), *U. sulphureus* primarily preys on cephalopods, with cephalopods contributing 23% to its diet, followed by zooplankton (16%) and POM (14%). In contrast, *U. tragula* had a more balanced diet, with macrurans, POM, zooplankton, and brachyurans each contributing approximately 13% to its food sources (Table 5).

**TABLE 4:** The carbon and nitrogen stable isotope ratios of *U. sulphureus* , *U. tragula* , and their potential food sources.

Catagory	Sample number	δ^13^C (‰)		δ^15^N (‰)	
		Range	Mean ± SD	Range	Mean ± SD
*U. sulphureus*	68	−19.53 to −17.83	−18.38 ± 0.35	12.45 to 14.52	13.31 ± 0.47
*U. tragula*	57	−17.33 to −13.70	−15.23 ± 0.96	14.46 to 16.25	15.36 ± 0.38
Brachyura	41	−19.45 to −11.80	−16.72 ± 1.32	10.13 to 15.32	12.56 ± 1.13
Cephalopoda	32	−19.57 to −15.80	−17.85 ± 1.03	9.03 to 15.25	11.99 ± 1.40
Gastropoda	16	−19.67 to −16.30	−17.54 ± 0.97	7.26 to 11.50	8.95 ± 1.28
Macrura	51	−18.63 to −13.48	−17.08 ± 0.95	9.18 to 14.93	11.71 ± 1.66
Phytoplankton	10	−22.66 to −20.34	−21.63 ± 0.79	4.46 to 7.41	6.10 ± 0.87
POM	12	−26.32 to −22.87	−24.88 ± 1.04	2.55 to 4.53	3.33 ± 0.55
SOM	21	−25.70 to −8.16	−16.81 ± 5.49	2.97 to 3.62	3.22 ± 0.20
Stomatopoda	35	−18.40 to −15.92	−17.44 ± 0.80	10.21 to 15.41	12.25 ± 1.47
Zooplankton	29	−25.68 to −19.50	−22.22 ± 1.79	5.37 to 13.65	8.13 ± 1.75

**TABLE 5:** MixSIAR analysis results on the contribution of food sources to *U. sulphureus* and *U. tragula* .

Catagory	*U. sulphureus*		*U. tragula*	
	Mean	SD	Mean	SD
Brachyura	0.101	0.107	0.125	0.116
Cephalopoda	0.226	0.138	0.104	0.110
Gastropoda	0.024	0.027	0.085	0.096
Macrura	0.118	0.092	0.130	0.116
Phytoplankton	0.075	0.050	0.096	0.098
POM	0.141	0.105	0.129	0.101
SOM	0.064	0.050	0.093	0.084
Stomatopoda	0.094	0.066	0.112	0.104
Zooplankton	0.157	0.056	0.127	0.101

**FIGURE 3:**
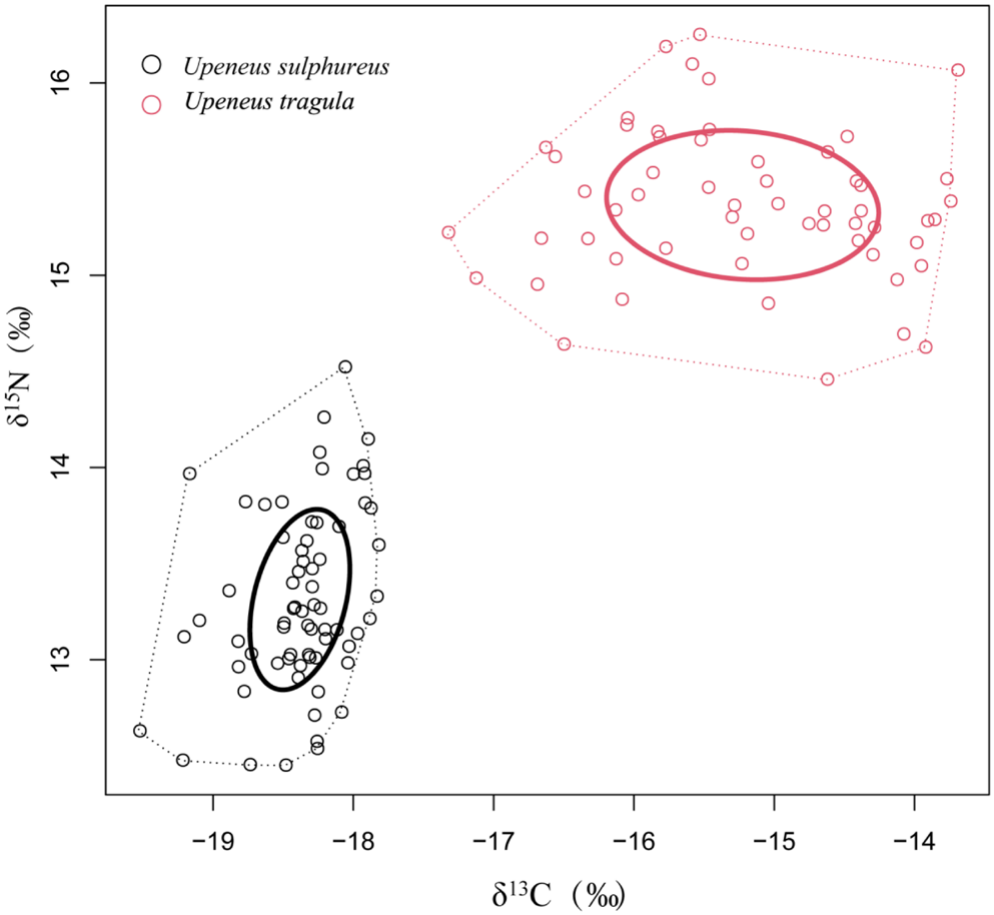
Overlap of *U. sulphureus* and *U. tragula* in the ecological niche.

Based on carbon and nitrogen stable isotopes, the trophic niche analysis of *U. sulphureus* and *U. tragula* revealed the following results (Table 6): For *U. sulphureus* , the trophic diversity level (CD) was 0.50, the horizontal niche width (CR) was 1.70, the vertical niche width (NR) was 2.07, the overall density (MNND) was 0.10, the trophic niche distribution range (SDNND) was 0.09, the core niche (SEAc) was 0.49, and the total niche area (TA) was 2.47. For *U. tragula* , the trophic diversity level (CD) was 0.92, the horizontal niche width (CR) was 3.63, the vertical niche width (NR) was 1.79, the overall density (MNND) was 0.16, the trophic niche distribution range (SDNND) was 0.11, the core niche (SEAc) was 1.18, and the total niche area (TA) was 4.75. The trophic niches of *U. sulphureus* and *U. tragula* did not overlap, indicating clear trophic niche separation between the two species (Figure 3).

**TABLE 6:** SIBER analysis results on the trophic niche overlap of *U. sulphureus* and *U. tragula* .

Specise	CD	CR	NR	MNND	SDNND	SEAc	TA
*U. sulphureus*	0.50	1.70	2.07	0.10	0.09	0.49	2.47
*U. tragula*	0.92	3.63	1.79	0.16	0.11	1.18	4.75

Abbreviations: CD, centroid distance; CR, δ^13^C range; MNND, mean nearest neighbor distance; NR, δ^15^N range; SDNND, standard deviation of nearest neighbor distance; SEAc, standard ellipse area; TA, total convex hull area.

### 3.3 Analysis of the Relationship Between Functional Niche and Trophic Niche

**FIGURE 4:**
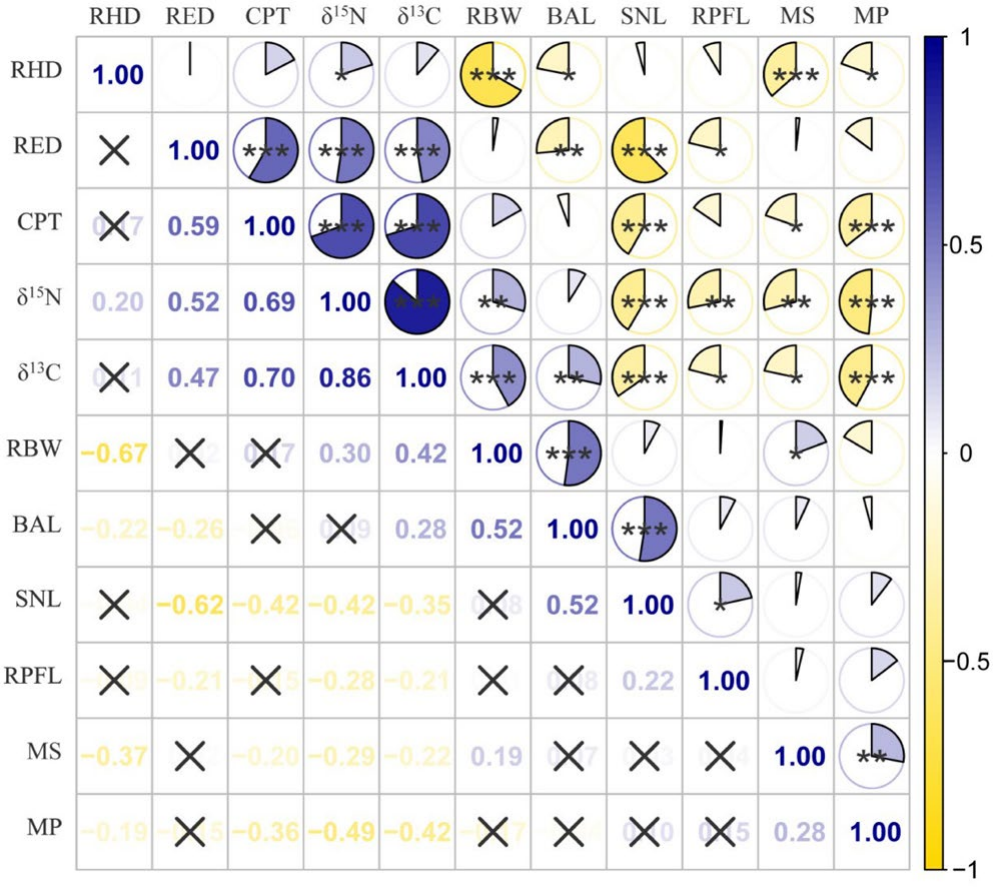
Spearman's correlation between ecomorphological traits and stable isotope. Values show Pearson correlation coefficients (−1 to 1). Red indicates positive correlation, blue indicates negative correlation. *p < 0.05, **p < 0.01, ***p < 0.001; X indicates no statistical significance (*p* ≥ 0.05).

Spearman's rank correlation analysis (Figure 4) revealed significant associations between morphological traits and stable isotope ratios in the study subjects (*n* = 104), with BD/SL, ED/HD, and CFD/CPD showing strong positive correlations with both δ^15^N (*p* = 2.17 × 10^−3^, 1.16 × 10^−8^, and 2.88 × 10^−16^, respectively) and δ^13^C (*p* = 8.98 × 10^−6^, 3.65 × 10^−7^, and 8.34 × 10^−17^, respectively; all *p* < 0.001), while MD/MW (δ^15^N: *p* = 2.77 × 10^−3^; δ^13^C: *p* = 2.73 × 10^−2^), MO/HD (δ^15^N: *p* = 1.76 × 10^−7^; δ^13^C: *p* = 8.20 × 10^−6^), PFL/SL (δ^15^N: *p* = 3.89 × 10^−3^; δ^13^C: *p* = 3.21 × 10^−2^), and EHL (δ^15^N: *p* = 1.16 × 10^−5^; δ^13^C: *p* = 2.94 × 10^−4^) exhibited significant negative correlations.

## 4 Discussion

### 4.1 Morphological Characteristics and Functional Niches

The functional diversity of fish communities can be measured through interspecific variation (assuming intraspecific variation is negligible) (Zhao et al. 2017) or morphological traits (such as body size, caudal peduncle length, and pectoral fin width) (De Bello et al. 2011; Villéger et al. 2017). By comparing the morphological traits of *U. sulphureus* and *U. tragula* , significant differences were found in their caudal peduncle, oral gape, barbel length, pectoral fins, and snout length (*p <* 0.05).

The study found that *U. sulphureus* exhibits typical benthic–pelagic transitional morphology. It has a robust body, a narrow caudal peduncle, and long pectoral fins. These traits suggest slower swimming and flexible movement across vertical habitats (Gatz Jr. 1979; Page and Swofford 1984; Webb 1988). Its large oral gape and short barbels indicate a suction‐feeding strategy (Gatz Jr. 1979). This species likely targets small pelagic fishes or planktonic invertebrates rather than relying on barbel‐mediated benthic probing (Uiblein 1991). In contrast, *U. tragula* displays specialized benthic foraging adaptations. Its streamlined body and wider caudal peduncle enhance sustained swimming, facilitating long‐distance searches over sandy–muddy substrates (Giammona 2021; Han et al. 2020; Webb 1988). Larger eyes and elongated barbels suggest a combined visual–tactile hunting strategy. This allows efficient detection of concealed benthic prey, such as polychaetes and small crustaceans (Uiblein and Winkler 1994). Uiblein et al. (1998) noted that species with long and thick barbels ( *Upeneus sundaicus* ) exhibit more benthic foraging behavior. Long barbels may possess more taste buds, enabling prey detection through chemosensory mechanisms (McCormick 1993), and may also assist in capturing prey from crevices in hard substrates (Uiblein 1991). Goatfish can also balance foraging and predator avoidance by adjusting barbel structure and maintaining specific body postures, demonstrating their adaptability to specific environments (Gosline 1984).

The low functional niche overlap (27.91%) between *U. sulphureus* and *U. tragula* may reflect similarities in certain resource utilization strategies but differences in ecological adaptation and resource exploitation (Aneesh Kumar et al. 2017). *U. tragula* exhibits higher functional richness, indicating a broader capacity for resource utilization. The observed differences in feeding and locomotion‐related functional traits between the two species could promote niche differentiation and facilitate their coexistence.

### 4.2 Potential Food Sources and Nutrient Niches

The morphological traits of goatfish are closely related to their ecological functions, reflected in habitat selection, dietary characteristics, and their functional roles within ecosystems. Based on reports of stomach contents (Hiatt and Strasburg 1960; Hobson 1974), goatfish prey on a variety of small and medium‐sized animals, showing no specialized feeding preferences, and are opportunistic carnivorous predators that impact benthic fauna (Chérif et al. 2011). This study analyzed the potential food sources of *U. sulphureus* and *U. tragula* , finding significant variation in the dietary composition of *U. sulphureus* , while the contributions of different food types in *U. tragula* were relatively uniform. Notably, cephalopods constituted a higher proportion in the diet of *U. sulphureus* , likely due to its high mobility and swallowing capacity, enabling effective capture of agile cephalopods (Villanueva et al. 2017). *U. tragula* appears to exhibit different foraging strategies compared to *U. sulphureus* , more frequently using barbels to probe the substrate and relying on visual predation (Gosline 1984) to capture invertebrates buried in the substrate (Sabrah and El‐Ganainy 2009). Hiatt and Strasburg (1960) noted that *Upeneus arge* does not use barbels to probe the sand like other goatfish but instead directly searches for benthic crustaceans, which may allow it to swim faster than other goatfish. Different feeding preferences are closely linked to the species' habitat, locomotion, and specific predation strategies (Labropoulou and Papadopoulou‐Smith 1999), and these factors collectively shape resource utilization patterns, influencing dietary choices and ecological adaptability (Webb 1984).

The trophic niche of *U. sulphureus* is relatively narrow, with lower trophic diversity (CD), indicating a more specialized selection of food resources. In contrast, *U. tragula* exhibits higher trophic diversity (CD), indicating a broader range of food sources and greater adaptability. The SEAc value of *U. tragula* is significantly higher than that of *U. sulphureus* , suggesting a larger core niche and a more diverse range of food sources. The larger TA value of *U. tragula* indicates that it occupies a larger trophic niche space within the ecosystem, potentially reflecting stronger resource utilization capabilities. *U. sulphureus* and *U. tragula* exhibit significant differentiation in their trophic niches, and this niche separation effectively reduces interspecific competition between the two species, thereby promoting coexistence (Matich et al. 2017; Ornelas‐García et al. 2018).

### 4.3 The Relationship Between Functional Traits and δ^13^C, δ^15^ Ratios

Analysis indicates a strong association between trophic niche shifts and multiple functional traits. *U. sulphureus* and *U. tragula* exhibit highly significant differences in δ^13^C (*p* < 0.001) and δ^15^N (*p* < 0.001) values. Specifically, higher δ^15^N and δ^13^C values are significantly positively correlated with body width, eye diameter, and caudal peduncle length, but negatively correlated with oral gape shape, oral gape position, pectoral fin length, and snout length. *U. tragula* , with higher δ^15^N, displays longer barbels, larger eyes, a broader caudal peduncle, and a streamlined body, traits associated with foraging‐related locomotion, primarily feeding on small benthic invertebrates, resulting in higher δ^15^N values. Research by Fitzgerald et al. (2017) further confirms that in benthic‐feeding loricariid fish, caudal peduncle length, fin length, and body width are strongly correlated with δ^15^N.

*U. sulphureus* has lower δ^13^C values, characterized by a large oral gape, a long snout, and long pectoral fins, which may indicate traits related to food acquisition. *U. sulphureus* can use its long snout and mouth to excavate and feed on cephalopods (Lukoschek and McCormick 2001). Mittelheiser et al. (2022) found that species with longer snouts ( *Parupeneus barberinus* ) typically have lower δ^13^C values, associated with their foraging behavior in deeper substrate layers or crevices. In contrast, species with shorter snouts ( *Mulloidichthys flavolineatus* ) exhibit higher δ^13^C values, consistent with their tendency to forage by digging in shallow, soft substrates. Fish with strong swimming abilities may forage across different water layers, and their δ^13^C values may reflect their broad habitat range. In contrast, benthic fish typically have higher δ^13^C values, reflecting their reliance on benthic food chains. These morphological differences may be closely linked to their distinct feeding strategies and prey type preferences, thereby influencing their distribution patterns within the trophic niche.

Although *U. sulphureus* and *U. tragula* exhibit some overlap in their functional niches (27.91%), niche separation persists, likely due to differences in food acquisition strategies, morphological traits, and trophic levels. Niche separation helps reduce interspecific competition, enabling both species to coexist in similar environments while utilizing different resources (Reyes‐Puig et al. 2024). This phenomenon is ecologically significant, as it demonstrates how species avoid direct resource competition through divergent adaptive strategies.

## 5 Conclusions

The significant niche differentiation was observed between *U. sulphureus* and *U. tragula* (*p* < 0.05). *U. sulphureus* exhibits a robust body, longer pectoral fins and snout, lower δ^13^C (−18.38‰ ± 0.35‰) and δ^15^N (13.31‰ ± 0.47‰) values, and primarily feeds on cephalopods. In contrast, *U. tragula* has a streamlined body, larger eye diameter, longer barbels, higher δ^13^C (−15.23‰ ± 0.96‰) and δ^15^N (15.36‰ ± 0.38‰) values, and preys on small benthic invertebrates. Functional niche analysis revealed that *U. tragula* had significantly higher functional richness (22.33%) than *U. sulphureus* (11.64%), with low niche overlap (27.91%), indicating distinct resource partitioning. Trophic niche metrics further confirmed that *U. tragula* exhibited greater trophic diversity (CD) and core niche breadth (SEAc), suggesting broader resource utilization adaptability. These ecomorphological adaptations imply that the co‐occurring *U. sulphureus* and *U. tragula* in the Beibu Gulf likely employ different locomotion modes and exploit distinct resource dimensions within the same habitat. The highly developed hyoid barbels may serve as a key evolutionary trait enabling their ecological specialization in goatfishes.

In contemporary ecomorphological research, the focus has gradually shifted toward exploring the complex relationships between species' morphology and their ecological functions (Andrew Barr 2018). These studies not only examine how species adapt to changing environmental conditions but also delve into how morphological structures influence their roles and resource utilization strategies within ecosystems (França and Severi 2022; Mittelheiser et al. 2022; Zhao et al. 2014). Particularly in the context of global changes posing challenges to biodiversity and ecosystem stability, ecomorphology provides a critical perspective for understanding and predicting species' responses and adaptability (Albouy et al. 2011). To more rigorously evaluate the validity of our ecomorphological interpretations, it is essential to collect additional data on the behavior and ecology of these species.

## Author Contributions

**Jiawei Fu:** data curation (lead), formal analysis (lead), investigation (equal), methodology (equal), software (lead), writing – original draft (lead), writing – review and editing (lead). **Xiaodong Yang:** data curation (equal), methodology (equal), writing – review and editing (lead). **Konglan Luo:** methodology (equal), software (equal), writing – review and editing (equal). **Zhisen Luo:** investigation (equal), writing – review and editing (equal). **Jingxi Wang:** investigation (equal), writing – review and editing (equal). **Bin Kang:** conceptualization (equal), methodology (equal), project administration (lead). **Xiongbo He:** data curation (equal), methodology (equal), project administration (lead), software (equal), writing – review and editing (lead). **Yunrong Yan:** conceptualization (equal), funding acquisition (equal), methodology (equal), project administration (lead), writing – review and editing (lead).

## Conflicts of Interest

The authors declare no conflicts of interest.

## Data Availability

The datasets supporting the findings of this research, have been deposited in the Dryad repository. The data are publicly available. Data to support this study is available from the https://doi.org/10.5061/dryad.j9kd51cqc.
